# The Veterinary Association of Manitoba

**Published:** 1894-12

**Authors:** W. J. Hinman

**Affiliations:** Registrar


### THE VETERINARY ASSOCIATION OF MANITOBA.

Under the authority of Secs. 18, 19, 20, 22 and 26 of the Veterinary Association Act, 1890 (53 Vic., Chap. 60), the following persons ONLY are entitled to practice as Veterinary Surgeons in the Province of Manitoba, or to collect fees for the service rendered as such:

| | |
|---|---|
| Atkinson, J. C., | Winnipeg. |
| Baker, G. P., | Binscarth. |
| Borradil, A., | McGregor. |
| Coote, H. L., | Minnedosa. |
| Coxe, S. A., | Brandon. |
| Dunbar, W. A., | Winnipeg. |
| Dann, Joseph, | Deloraine. |
| Fisher, John Frederick, | Brandon. |
| Graham, N., | Manitou. |
| Hatton, J., | Pipestone. |
| Hinman, Willet, J., | Winnipeg. |
| Henderson, W. S., | Carberry. |
| Irwin, John James, | Stonewall. |
| Little, Charles, | Winnipeg. |
| Little, William, | Boissevain. |
| McFadden, D. H., | Emerson. |
| McGillivray, J. D., | Manitou. |
| McMillan, Adam, | Oak Lake. |
| McNaught, David, | Rapid City. |
| Marshall, H. A., | Glenboro. |
| Menteith, H. E., | Killarney. |
| Morrison, Wm. McLeod, | Glenboro. |
| Murray, Geo. P., | Morden.. |
| MaLoughrey, A., | Elkhorn |
| Rutherford, John Gunion, | Portage in Prairie. |
| Rombough, M. B., | Morden. |
| Shoults, Wm. A., | Gladstone. |
| Smith, Henry D., | Winnipeg. |
| Spiers, John, | Virden. |
| Sweet, T. J., | Morden. |
| Smith, W. H., | Carman. |
| Taylor, William Ralph, | Portage la Prairie. |
| Thompson. S. J., | Carberry. |
| Torrance, Frederick, | Brandon. |
| Walker, J. St. Clair, | Boissevain. |
| Wood, S. H., | Selkirk. |
| Young, M., | Manitou. |

The practice of the veterinary profession in Manitoba by any other person is in direct contravention of the statute and renders him liable for prosecution.

W. J. HINMAN, Registrar.

